# Poster Session I - A86 REAL LIFE DIAGNOSTIC ACCURACY OF HIGH-RESOLUTION ANORECTAL MANOMETRY FOR THE DIAGNOSIS OF HIRSCHSPRUNG’S DISEASE IN CHILDREN

**DOI:** 10.1093/jcag/gwaf042.086

**Published:** 2026-02-13

**Authors:** M Awouters, C Faure, A Ann

**Affiliations:** Pediatric gastroenterology, Centre Hospitalier Universitaire Sainte-Justine, Montreal, QC, Canada; Pediatric gastroenterology, Centre Hospitalier Universitaire Sainte-Justine, Montreal, QC, Canada; Pediatric gastroenterology, Centre Hospitalier Universitaire Sainte-Justine, Montreal, QC, Canada

## Abstract

**Background:**

High-Resolution Anorectal Manometry (HRARM) increasingly replaces conventional ARM for the assessment of the rectoanal inhibitory reflex (RAIR) for diagnosing Hirschsprung’s disease (HD). However, historic studies only included patients with a clinical suspicion of HD and its diagnostic accuracy in a broader pediatric population with severe constipation remains unclear.

**Aims:**

We aimed to evaluate the diagnostic accuracy of HRARM in diagnosing HD in children with severe constipation.

**Methods:**

We retrospectively analyzed HRARM studies performed for severe constipation performed at CHU Sainte-Justine, Montreal, between April 2016 and April 2024. Patients were grouped by age: < 6 months old, 6-12 months old and ≥ 12 months old. RAIR was deemed positive when both reproducible and proportional to balloon volume.

**Results:**

Among 702 patients, eleven (1.6%) were diagnosed with ultrashort or short-segment HD. Overall HRARM sensitivity was 82%, specificity 91%, PPV 13%, and NPV 99.6% (Table 1). Sensitivity increased with age, while specificity and PPV decreased. Two diagnoses were initially missed, both in children < 6 months old. False negatives occurred in infants <6 months due to agitation or stool evacuation. False positives were linked to agitation and sphincter contractions in infants, and megarectum or anxiety in older children. Anal resting pressure did not differ between HD and non-HD patients.

**Conclusions:**

HRARM is a valuable screening tool for HD in children with severe constipation, especially given its high NPV in those >1 year. We recommend anorectal biopsy only in patients with absent or equivocal RAIR if supported by a suggestive clinical history. In infants <6 months, results should be interpreted cautiously, and further investigation remains warranted when HD is clinically suspected despite a present RAIR.

**Funding Agencies:**

None

